# Explaining Conflicting Results in Research on the Heterogeneous Effects of Parental Separation on Children’s Educational Attainment According to Social Background

**DOI:** 10.1007/s10680-017-9417-5

**Published:** 2017-03-20

**Authors:** Fabrizio Bernardi, Diederik Boertien

**Affiliations:** 1 European University Institute, Fiesole, Italy; 2 Centre d’Estudis Demogràfics, Barcelona, Spain

**Keywords:** Divorce, Education, Family, Inequality

## Abstract

In recent years, researchers have become increasingly interested in how the effects of parental separation on children’s educational attainment vary with social background. On the one hand, parents with more resources might be better able to prevent possible adverse events like separation to affect their children’s outcomes. On the other hand, children from higher social backgrounds might have more resources to lose from a parental separation. A wide range of empirical studies on the issue have come to inconsistent conclusions, with support found for both perspectives. The aim of this paper is to monitor the influence of methodological and operational choices on the different results observed across studies. We focus on aspects such as the operationalization of key variables, the measurement of inequality in absolute and relative terms and the different strategies used to address endogeneity. We study the effects of parental separation on educational attainment for a cohort of British children born in 1970 and find that conclusions change depending on whether social background is measured using the mother’s or father’s characteristics and whether relative or absolute differences between groups are considered. Results are relatively insensitive to the operationalization of dependent variables and the treatment of missing data. When using data from Understanding Society instead of the British Cohort Study, results also did not change. We reflect on how these findings can explain the contradictory results from earlier studies on the topic, and how heterogeneity in the effects of parental separation by socio-economic background should be interpreted.

In recent years, researchers have become increasingly interested in how the effects of parental separation on child outcomes vary with social background (Albertini and Dronkers 2009; Augustine 2014; Bernardi and Boertien 2016; Bernardi and Radl 2014; Biblarz and Raferty 1993, 1999; Biblarz et al. 1997; Cavanagh and Huston 2006; Elliott and Richards 1991; Fischer 2007; Jonsson and Gähler 1997; Mandemakers and Kalmijn 2014; Martin 2012; McLanahan and Sandefur 1994). From a theoretical perspective, it is unclear whether one should expect the negative consequences of parental separation on child outcomes to be greater or smaller for socio-economically advantaged children. On the one hand, socio-economically advantaged parents could employ their resources to prevent adverse events like separation from affecting their children’s outcomes. On the other hand, advantaged parents might experience greater absolute losses in financial resources following separation, and their children might lose more from weakened contact with their father or from changes in parental monitoring. Despite several papers addressing the empirical question of whether children from distinct social backgrounds experience different ‘separation penalties’, no clear answer can yet be given. Some studies conclude that greater adverse effects are observed in children from higher socio-economic backgrounds (Bernardi and Radl 2014; Biblarz and Raferty 1993; Martin 2012; McLanahan and Sandefur 1994), whereas others come to the opposite conclusion (Albertini and Dronkers 2009; Augustine 2014; Cavanagh and Huston 2006; Grätz 2015; Mandemakers and Kalmijn 2014).

There are several possible reasons why the aforementioned studies have not produced consistent results. First, they refer to different countries, time periods and/or cohorts. A number of *institutional factors* that differ across time and space might moderate or reinforce the impact of parental separation, social background and their interplay on children’s outcomes. For instance, the presence of laws on joint custody in the case of parental separation and the societal characteristics affecting the outcome studied (e.g. the educational system in the case of educational attainment) are likely to cause variation in the effects of parental separation. Second, the existing studies consider different *child outcomes*, ranging from cognitive and non-cognitive skills, to income and educational and occupational attainment. It is likely that the underlying mechanisms that produce heterogeneity in the effects of parental separation vary in importance depending on the outcome studied. Third, results might vary across studies because of *methodological and operational aspects*. For instance, results can depend on choices made regarding the operationalization of key variables, the measurement of inequality in terms of absolute or relative differences, and the extent to which they account for possible endogeneity biases. A crucial issue in this respect is that selection into parental separation based on unobserved variables is likely to differ across countries and time periods/cohorts.

In this paper, we concentrate on the latter source of variability in results, i.e. sources related to the methodological and operational choices of studies. We aim to underline the importance of this matter by showing how researchers can make methodological choices that lead to different results, even when the same country, period and child outcome are studied. In this paper, we keep these factors constant by limiting our analysis to the educational attainment of the respondents of the British Cohort Study (BCS) born in 1970. For this birth cohort, we show how the operationalization of variables and the measurement of inequalities in absolute or relative terms lead to different conclusions regarding patterns of heterogeneity in the effects of parental separation. We also discuss how the way in which endogeneity is addressed (or not) might affect results.

The paper is organized as follows. We start with a discussion of how parental separation might affect children’s outcomes differently according to social background, followed by a review of the results from recent studies on the topic. We proceed by giving an overview of how methodological and operational choices might underlie part of the contradictory results found in these studies. In our empirical section, we use the 1970 British Cohort Study to examine how results vary based on different ways of operationalizing parental background, family situation and educational attainment. Subsequently, we employ different strategies to deal with endogeneity issues, such as pre-separation controls and a so-called ‘placebo test’. Finally, we replicate our analysis for a different data set (Understanding Society), and look at the influence of reporting results in terms of relative versus absolute differences (e.g. odds ratios versus absolute differences in predicted probabilities). We conclude by giving a substantive interpretation to the different findings and by providing some suggestions for future research.

## Literature Review

Research addressing heterogeneity in the effects of parental separation on child outcomes can be traced back to studies on social mobility which argued that separation could possibly increase intergenerational mobility (Biblarz and Raferty 1993, 1999; Coleman 1988). These studies suggested that separation constrains families and therewith complicates the transmission of human capital from parents to children. Given that the amount of human capital transmitted is hypothesized to be higher among socio-economically advantaged families, the consequences of a weakened transmission are greater for their children than for less advantaged children. These initial studies indeed found intergenerational mobility to be higher within separated families compared to non-separated families in the USA (Biblarz and Raferty 1993, 1999; Coleman 1988).

This perspective contrasts with another branch of the social stratification literature that focused on how adverse events, in general, affect children differently depending on their social background. These studies looked at the effects of disadvantages within the educational system, such as grade retention or being born in an ‘unfavourable month’ on later child outcomes (Bernardi 2012, 2014; Bernardi and Grätz 2015). Empirical results point to the existence of ‘compensatory’ effects where parents from higher social classes are more able to (partly) prevent their children from being affected by such disadvantages.

From a theoretical perspective, one could thus expect the answer to the question of who is affected most by parental separation—children of advantaged or disadvantaged social backgrounds—to go either way. Empirical results looking directly at heterogeneity in the effects of separation according to social background are mixed, with either possibility finding support in some of the studies published. Table 1 gives an overview of the main characteristics of 13 recent studies on the topic. Around half of the studies find children of higher social backgrounds to be affected more by parental separation, a little less than half support a mixed pattern differentiated by the gender of the parent (in italics), and two studies find greater effects on children of lower social backgrounds (in boldface). It is therefore not possible to give a straightforward answer to the question of how the effects of parental separation differ with social background and, hence, which of the two theoretical perspectives should be supported.

**Table 1:** Studies looking at the heterogeneity in the effects of parental separation on child outcomes

Article	Country and birth cohort	Outcome variable	Parental resources	Empirical strategy	Absolute versus relative	Family structure	Result
Final educational and occupational attainment							
Biblarz and Raferty (1993)	US, representative survey 1973	Occupational status of men	Paternal occupation	Cross-sectional	Relative	Living with both parents until age 16	Smaller intergenerational transmission for children of separated parents
McLanahan and Sandefur (1994)	US	School drop-out	Parental college education combined	Cross-sectional	Relative	Living with both parents	If both parents went to college effects of parental absence are more negative
Fischer (2007)	Netherlands, representative survey 1998/2000	Educational and occupational attainment	Father’s and mother’s education and occupations	Cross-sectional	Absolute	Divorce before age 18	*Most negative effects of parental divorce when mother’s education is low and father’s is high for both educational attainment and occupational status at age 30. For educational attainment at age 15 same results, but weaker*
Albertini and Dronkers (2009)	Italy (1950–1985)	Educational attainment	Mother’s education	Cross-sectional	Relative	All nonstandard forms together	*Parental separation effect non*-*existent for children with higher-educated mothers*
Martin (2012)	US (1974)	Grades, test scores, educational attainment	Parents’ average years of education	Cross-sectional	Both	All nonstandard forms separate	On all outcomes high-SES children have a higher separation penalty. The same for other nonstandard family forms
Bernardi and Boertien (2016)	UK (1970)	Educational attainment	Father’s and mother’s education	Pre-separation controls	Absolute	Separation between ages 5 and 16	Children affected most negatively if both parents have at least more than high school education
Bernardi and Radl (2014)	Cross-national (1945–1984)	Educational attainment	Highest level of education among parents	Cross-sectional	Absolute	Parental separation before age 18	Higher separation penalty for high-SES children. Not the case in early tracking countries (10–12 years)
Educational performance							
Jonsson and Gähler (1997)	Sweden (1972–1976)	School track placement	Mother’s and father’s education	Pre-separation controls	Absolute	All nonstandard forms separate	*Largest negative effects of parental separation when mother’s education is low and father’s education high. See p.190*
Augustine (2014)	US (1991)	Mathematics and reading skills	Mother’s years of schooling	Growth curves	Absolute	All nonstandard forms together	*Children from higher-educated mothers less negatively affected*
Grätz (2015)	Germany (1983–1994)	Grades, track placement, re-taking year	Parents’ combined level of education	Sibling fixed-effect models	Absolute	Not living with both parents all of childhood	**Cross-section: higher-educated affected more negatively as regards GPA. Sibling models: Higher-educated affected less negatively, also when splitting by gender of parent, both GPA and track placement**
Behavioural problems							
Cavanagh and Huston (2006)	US (1991)	Externalizing behaviour	Income and home resources to stimulate child development	Longitudinal	Absolute	Number of family transitions	**Income and home resources reduce negative effect of parental separation on behaviour**
Elliott and Richards (1991)	UK (1958)	Behavioural problems, test scores, educational attainment	Paternal social class	Longitudinal	Absolute	Ceasing to live with both parents between ages 7 and 16	More negative effects of separation on reading test scores for non-manual fathers’ children. No statistically significant interaction effects found for educational attainment and behaviour
Mandemakers and Kalmijn (2014)	UK (1970)	Behavioural problems and test scores	Mother’s and father’s education	Repeated measures of well-being	Absolute	Separation between ages 5 and 10	*Mother’s education significantly dampens separation effect for well*-*being. Father’s education strengthens separation effect*

Results in boldface congruent with greater effects on children of lower social backgrounds

Results in italics support a mixed pattern according to the gender of the parent whose resources are considered

Only a handful of studies have examined empirically the mechanisms that produce heterogeneity in the effects of parental separation. A recent study on educational attainment in the UK (Bernardi and Boertien 2016), which found greater effects on children from advantaged socio-economic backgrounds, pointed at the role of choices in the educational system and financial resources. Children from advantaged social backgrounds lose more family income following separation and, in addition, losses in family income are more consequential to their educational attainment. Family income appeared to be less related to educational attainment for children from disadvantaged families, possibly because family incomes of lower-educated parents are too low to invest in the education of children in the first place.

A US study on educational attainment (Martin 2012), in line with these claims, shows that heterogeneity in effects according to social background can be partially explained by lower educational expectations on the part of parents, less engagement of children in structured leisure activities, and less involvement of parents in their children’s schools after separation. Studies that found children from socio-economically disadvantaged backgrounds to be affected more by parental separation did not look directly at the mechanisms underlying their results.

## Substantive Reasons for Conflicting Results

As discussed in more detail in the introduction to this special issue, there are several possible substantive reasons for inconsistent findings across studies on the effects of parental separation. First, studies have focused on different child outcomes ranging from behavioural problems to cognitive skills and educational and occupational attainment. Parental separation is negatively associated with all of these outcomes, but how its effects are conditioned by social background appears to differ according to the outcome studied, i.e. the interaction effect of parental separation with parental resources differs depending on the outcome being examined. The overview given in Table 1 suggests that studies on educational and occupational attainment in general point to greater effects for socio-economically advantaged children, whereas the results appear more mixed or opposing for cognitive skills and behavioural problems Cavanagh and Huston 2006; Elliott and Richards 1991; Mandemakers and Kalmijn 2014).

Second, studies are conducted in countries and time periods that differ on a number of institutional dimensions that affect the impact of parental separation and social background on children’s outcomes. For instance, Bernardi and Radl (2014) found the heterogeneity in the effects of parental separation on educational attainment to differ across countries, using harmonized measures and methods for each country. The effects of parental separation have generally been attributed to factors such as losses in income, family conflict and changes in parental styles (Amato 2010). The extent to which such mechanisms are set into motion is likely to differ across contexts. For instance, whether children have less access to economic resources following separation can depend on the cost of divorce, child maintenance regulations and welfare state policies supporting single mothers. Depending on the outcome studied, institutional factors will also matter for the way in which a specific desirable child outcome is attained. For example, when studying educational attainment the extent to which high tuition fees complicates access to tertiary education can moderate the importance of changes in income following parental separation.

The research in this area is, however, still at an early stage. A systematic review of how the interplay between parental separation and socio-economic background might vary depending on institutional factors and the outcomes considered is therefore not possible. Here, we explore how different methodological and operational choices might affect results. As we will show, such choices could be highly consequential for the conclusions drawn and create an extra layer of complexity to the body of research, preventing the accumulation of knowledge across studies.

## Methodological and Operational Choices Affecting Results

### Dealing with Endogeneity

An initial, important difference across studies is how they deal with endogeneity between parental separation and children’s educational outcomes. Associations between parental separation and children’s educational attainment could reflect unobserved differences between families that preexisted an eventual parental separation (e.g. family conflict). In such cases, these unobserved characteristics could be responsible for differences in final attainment rather than separation itself (Bernardi and Martínez-Pastor 2011). When studying heterogeneity in the effects of parental separation, an additional layer of complexity is added, as such selection into parental separation could differ by socio-economic background. Differences in parental separation penalties according to social background could be due to differential selection into parental separation within social groups. Studies that have attempted to control for such pre-separation characteristics normally found results to be robust (Bernardi and Boertien 2016; Mandemakers and Kalmijn 2014; Martin 2012). When relying on a strategy of observable controls, key variables to include would be factors simultaneously determining separation and educational in general (i.e. family conflict, economic resources and parenting styles pre-separation) and factors that affect the differential selection into divorce by socio-economic background.^1^

The majority of studies reviewed in Table 1 rely on controlling for observable characteristics. Two studies, however, have employed more rigorous methods to deal with endogeneity. A study in Britain used a ‘placebo test’ (Bernardi and Boertien 2016). This test consisted of estimating the associations of parental separations taking place at time *t* with school performance and educational choices measured at *t* − *1,* i.e. the years immediately preceding the event of separation. If relatively stable preexisting differences between groups drive results, these differences should be visible already in the years before the event of separation. In this study, parental separations were not associated with outcomes preceding the separation. At the same time, parental separations were related to educational attainment after the separation event and had a greater effect on the outcomes of advantaged children. In this case, preexisting differences between groups did not produce (heterogeneity in the) associations between parental separation and school performance or educational choices.

In contrast, a study in Germany, which also looked at school performance, employed sibling fixed-effects models. Such models control for all family-invariant unobserved characteristics. These sibling fixed-effects models suggested that parental separation only affects the outcomes of children from socio-economically disadvantaged backgrounds. However, when these estimates were compared to ‘naïve’ estimates that did not control for these unobserved factors, the results were turned around, with advantaged children being affected more in terms of grades (Grätz 2015). In this case, unobserved heterogeneity did play a role. The importance of endogeneity could thus indeed differ across institutional contexts. However, one should also note that sibling fixed-effects models measure the effects of age at separation (Sigle-Rushton et al. 2014) or the differential response to parental separation according to birth order of the child rather than the experience of separation itself.

### Operationalization of Key Variables

Besides the general strategy used to account for possible endogeneity, another possible source of heterogeneity in the results is the operationalization of key variables. The basic model estimated in the papers reviewed consists of a child outcome as the dependent variable and two independent variables, namely parental separation and parental socio-economic background. The operationalization of both these independent variables varies considerably across studies (see Table 1). If a researcher has different options at her or his disposition to operationalize variables, the danger arises that the option selected is that which provides significant or desired results (Gelman and Loken 2014). In this paper, we aim to minimize this danger by documenting how different choices made regarding the operationalization of key variables influence substantive results.

An essential driver of difference in results appears to be whether one distinguishes between maternal and paternal resources when operationalizing social background. As displayed in Table 1, several of the studies reviewed find larger effects of parental separation where paternal resources are high, but maternal resources are low (in italics). The two studies that only looked at maternal resources found a general pattern of greater effects of parental separation on children with educated mothers (Albertini and Dronkers 2009; Augustine 2014). Given that normally the mother continues to live with her children following separation, this might not be surprising. Resources of the resident parent will still be accessible to children; therefore, the lower those resources, the harder it is to deal with the consequences of a separation. Resources of the nonresident parent, however, might be more complicated to access following separation. Therefore, the more resources the nonresident parent has, the more is lost.

Apart from deciding how to model the effects of both parents’ resources, the kind of parental resources one looks at could also influence results. Studies have used parental occupation, parental education, but also the presence of an educationally stimulating home environment (Cavanagh and Huston 2006). Looking at the results summarized in Table 1, studies using parental occupation or education do not seem to differ systematically from each other.

The second key independent variable is family structure. The main choice to be made here is how to categorize different kinds of ‘non-traditional family forms’. Some studies explicitly look at children who experienced a parental separation during childhood (Bernardi and Boertien 2016; Fischer 2007), whereas others lump all ‘nonstandard’ forms together based on whether children lived with both parents at a certain age (Albertini and Dronkers 2009; Augustine 2014). In the latter case, estimates express the effects of growing up with a single parent and thus include the effects of parental separation, parental death and of being born into a single-parent family. These situations differ in many respects from each other, and their effects on child outcomes are likely to vary. However, as shown in the overview of Table 1, no systematic differences seem to emerge between studies that lump all nonstandard family forms together and studies that look at parental separation explicitly. This is confirmed by a study that found uniform results when looking at different nonstandard groups separately (Martin 2012).

### Absolute Versus Relative Differences in Probabilities

Several decisions are made when selecting and analysing the data that can influence results (e.g. data quality, sample selection, how to treat missing data, estimation model). One particular choice merits special attention, namely whether the interaction effect of parental resources and family structure is reported and interpreted in absolute or relative terms. When dichotomous dependent variables are used, a choice is usually made between reporting results in a relative way using, for instance, odds ratios (Albertini and Dronkers 2009) or reporting absolute differences such as predicted probabilities (or coefficients from Linear Probability Models, Bernardi and Radl 2014; Bernardi and Boertien 2016). Such a choice is often made on methodological grounds (Mood 2010), but it has important substantive implications since one switches from relative to absolute measures of inequality (Buis 2008). This distinction is essential because it can lead to opposing interpretations of results in many cases. If baseline probabilities of attaining a certain outcome differ between groups, effects similar in size in absolute terms will be bigger in relative terms for the group with a lower baseline probability. Studies operating within the relative paradigm (e.g. using odds ratios) are therefore more likely to conclude that children from lower social backgrounds have higher separation penalties, whereas studies looking at absolute differences are more likely to conclude the opposite, given that the baseline probability of attaining better outcomes is normally higher for those from high-SES backgrounds.

## Data and Methods

In the empirical section of this paper, we look at the influence of methodological and operational choices made on estimates of heterogeneity in effects of parental separation on educational attainment in Britain. We use the 1970 British Cohort Study (BCS), a longitudinal panel survey which sampled a cohort of children born in a particular week in 1970 and interviewed them at different points in their lives. Britain has relatively elevated levels of economic inequality (Smeeding 2005) and, currently, relatively high single mother poverty (Del Boca 2003; Single mother poverty was closer to the average in the 1980s when the respondents were adolescents, Lewis 1997) compared to other European countries. Child support in Britain is determined based on disposable income and is required until the child turns 16, or 20 if in full-time education, but only a minority of nonresident fathers actually pay (or, in the 1980s, paid) child support to the mothers of their children (Ermisch and Pronzato 2008; Lewis 1997; Skinner and Davidson 2009). Financial resources might therefore play a relatively bigger role in our sample compared to samples from other contexts. For this birth cohort, parental separation was already more common among lower-educated mothers (Bernardi and Boertien 2016), which might make our analysis relatively susceptible to the influence of differential selection into separation by social background. Our sample size ended up being 10,254 children due to dropping variables with missing data on our dependent variable (educational attainment). Compared to the original 1970 selected sample, 60% was still present in 2000 and 54.7% provided information on all key variables. We will discuss the possible influence of attrition and missing data later. As separation could have different effects for children of migrant parents, we also performed a robustness check excluding children whose mothers were born outside of Britain (11% of cases), and results proved to be robust.

As a robustness check, we used the Understanding Society data to replicate the basic models of the analysis. We used the first wave of the survey to measure family background and looked at maximum educational attainment obtained in any of the four waves currently available. We restricted the sample to individuals born after World War II and before 1984 in order for them to have reached their late 20 s at the time of the survey. This left us with a sample size of 22,357 individuals. Given that we focused on the first wave of the survey, these numbers are not affected by attrition. At the same time, a certain number (17.4%) of respondents did not provide complete information on the relevant survey questions.

### Measures

For our analysis, we concentrated on one of the most studied outcomes in this body of literature: educational attainment. We employed the following measures: the age at which the respondent left full-time education, a dummy of whether the respondent attained tertiary education by age 30, and a dummy of whether the respondent continued in full-time post-compulsory secondary education after age 16.^2^

The following measures of parental resources were compared: the age at which parents left full-time education, a dummy of whether parents attained any qualifications beyond lower secondary education (more than ISCED 1-2), and a dummy of whether parents had a non-manual occupation. We created separate variables for the education of mothers and fathers and a ‘dominance’ measure of the highest value on these variables attained by either the mother or father. For the dummy variables, we looked at the different combinations of maternal and paternal resources, whereas for the continuous measure ‘age parents left full-time education’, the average of the mother and the father was calculated to create a joint measure.

For family structure, we employed the two most commonly used measures: whether the child lived with both biological parents at age 16, and whether the child experienced a parental separation before age 16. Given that the main outcome variable is measured at age 30, we assigned a separate value to the separation variable to children who experienced parental separation between ages 16 and 30 to prevent these cases from influencing results. The same was done for children who never lived with both of their parents. Therefore, in the analysis the comparison is always between children who continuously lived with both parents (who also did not separate before the child turned 30), and children who did experience separation during the given time frame. In additional analysis, we also looked at some post-separation characteristics (measured at age 16): whether the child lived with a step-parent, with the father (13.5% of separated cases) or the mother, and whether the absent parent currently pays child maintenance to the resident parent (37.2% of separated cases).

A common strategy to address, to some extent, endogeneity issues is to include pre-separation controls that could catch preexisting differences between children from separated and non-separated families. Suspects for differential selection into parental separation by social background are economic differences between households and family conflict (Amato and Hohmann-Marriott 2007; Boertien and Härkönen 2014). We aimed to include measures for both measured at age 5, as well as measures capturing overall preexisting differences in children’s abilities and behavioural problems. In this part of the analysis, we looked at the effects of separations that occurred between ages 5 and 16. Material resources were measured on a standardized scale based on variables measuring whether parents owned their home, whether the household had various kinds of durable goods, how their neighbourhood was rated, and how well equipped the house was, according to the interviewer (*α* = 0.72). Maternal psychological well-being, which is related to family conflict (Demo and Acock 1996; Kim and McKenry 2002), is based on 25 questions regarding psychological problems of the mother. Measures of cognitive ability and behavioural problems at age 5 were included to more generally control for pre-separation differences in the likelihood of achieving ‘successful’ outcomes. The measure of cognitive ability consists of three tests taken at age 5 (the ‘Human Figure Drawing Test’, the ‘Copying design test’, and the ‘English Picture Vocabulary Test’). Behavioural problems at age 5 were measured by a standardized scale based on 38 questions regarding behaviour, physical and psychological problems all answered by the mother.

Table 2 displays descriptive statistics for the sample and the variables used in the analysis. For the Understanding Society analysis, we used measures that were identical to those used for the British Cohort Study. However, the possibilities were restricted to using a dummy for attaining tertiary education as the dependent variable, a categorical measure for parental education which consisted of the possible combinations of whether the mother and/or father attained more than high school education, and whether the child experienced a parental separation before age 14.

**Table 2:** Descriptive statistics of the British Cohort Study 1970 sample used *N* = 10,254 and Understanding Society sample (*N* = 22,357)

	Average	SD	Min	Max
*British Cohort Study 1970 sample (N = 10,254)*				
Child outcomes				
Obtained tertiary education at age 30	0.27		0	1
Age left full-time education	17.7	3.0	14	30
Made transition to post-compulsory education, age 16	0.45		0	1
Parental resources				
Age mother left full-time education	16.0	2.22	10	25
Age father left full-time education	15.7	1.70	10	25
Neither parent has qualifications	0.36		0	1
Only mother has more than high school qual.	0.19		0	1
Only father has more than high school qual.	0.10		0	1
Both have more than high school qual.	0.35		0	1
Family structure				
% Experienced parental separation before Age 16	0.17		0	1
% Of children not living with both parents at age 16	0.21		0	1
*Understanding Society sample (N = 22,357)*				
Year of birth	1964.5	11.1	1945	1984
% Obtained tertiary education	0.41		0	1
Neither parent has qualifications	0.31		0	1
Only mother has more than high school qual.	0.13		0	1
Only father has more than high school qual.	0.09		0	1
Both have more than high school qual.	0.47		0	1
% Experienced parental separation before age 14	0.10		0	1

### Procedure

For the continuous dependent variables, we employed OLS regressions, and for the dichotomous dependent variables we estimated logistic regressions reporting odds ratios (OR), predicted probabilities and Linear Probability Models (LPM) with robust standard errors. Results were also compared based on different ways of treating missing data: listwise case deletion and multiple imputation of independent variables (cases with missing data on the dependent variable were used in the imputation equation, but not in the analysis).

The analysis commenced with a basic model explaining educational attainment. This was followed by an investigation of how sensitive these estimates were based on the choice and operationalization of dependent and independent variables. Subsequently, we decided upon the model best suited to our substantive purposes and looked at its robustness to using different estimation techniques and data. Finally, we also aimed to address issues of endogeneity by performing a ‘placebo test’ (Adda et al. 2011; Bernardi and Boertien 2016). The placebo test consisted of looking at the association between parental separations that took place between ages 17 and 19, having made the transition to post-compulsory education at age 16 (i.e. before the parental separations took place). If relatively stable unobserved differences between separated and non-separated families are responsible for the observed patterns of heterogeneity, differences in child outcomes should already be present in the period immediately preceding the parental separation. By looking at these ‘placebo’ separations, an estimate can be given of the association of unobserved characteristics with preexisting differences in child outcomes. All the code (for STATA) that has been used for data management and the analysis is available from the authors on request.

## Results

Table 3 provides an overview of different ways to estimate the effects of parental separation on educational attainment at age 30 according to parental background. The dependent variable in these models is whether the respondent attained tertiary education (1) or not (0) by age 30. Model 0 shows that children whose parents separated before age 16 were 11% points less likely to have attained tertiary education by age 30. This could be regarded as a substantial effect given that the overall probability for children whose parents never separated is 30%. The subsequent models in Table 3 illustrate how the age at which parents left full-time education conditions this penalty.

**Table 3:** Overview of separate LPM models explaining the attainment of tertiary education at age 30; *N* = 10,254

	Model 0		Model 1		Model 2		Model 3	
	Coef.	SE	Coef	SE	Coef.	SE	Coef.	SE
Parental Separation before age 16	−0.108**	0.01	−0.099**	0.01	−0.095**	0.01	−0.096**	0.01
Age mother left full-time education			0.079**	0.00			0.045**	0.00
Age father left full-time education					0.064**	0.00	0.043**	0.00
Parental separation × Age mother left education			−0.003	0.01			0.015	0.01
Parental separation × Age father left education					−0.017**	0.01	−0.021**	0.01
Constant	0.297**	0.01	0.291**	0.01	0.291**	0.01	0.290**	0.00

	Model 4		Model 5		Model 6		Model 7	
	Coef.	SE	Coef.	SE	Coef.	SE	Coef.	SE
Parental Separation before age 16					−0.094**	0.01	−0.093**	0.01
Parental separation and residing with mother	−0.091**	0.01						
Parental separation and residing with father	−0.048	0.04						
Parental separation and child maintenance paid			−0.075*	0.02				
Parental separation and no maintenance paid			−0.103**	0.02				
Average age parents left full-time education					0.088**	0.00		
Highest age parents left full-time education							0.068**	0.00
Parental separation × Age parents left education					−0.012	0.01	−0.014*	0.01
Age father left full-time education	0.065**	0.00	0.064**	0.00				
Parental separation residing w mother × Paternal education	−0.018*	0.01						
Parental separation residing w father × Paternal education	−0.001	0.02						
Parental separation maintenance paid × Paternal education			−0.018	0.01				
Parental separation no maintenance × Paternal education			−0.001	0.01				
Constant	0.291**	0.01	0.294**	0.01	0.290**	0.00	0.290**	0.00

† *p* < 0.10; * *p* < 0.05; ** *p* < 0.01; data from British Cohort Study 1970

Models 1 and 2 show the effects of maternal and paternal education separately and reveal that the separation penalty is bigger the later the father left full-time education. However, they also point out that children whose mothers left full-time education at a younger age have an equally high separation penalty as those whose mothers did so at a later age. In Model 3, both interaction terms are included at the same time^3^ and, as found in earlier research, we now see a positive interaction term for maternal education that is almost as large as the negative interaction with paternal education: though it is not statistically significant. Substantive conclusions would therefore be different depending on whether one chooses to focus on maternal or paternal resources. In the theoretical section, it was suggested that the differential importance of maternal and paternal resources exists because the father normally moves out of the household, and access to his resources therefore becomes less smooth after separation. Models 4 and 5 provide support for this suggestion by showing that the effect of parental separation does not differ by father’s education if children live with their father, or if the nonresident parent pays child maintenance. When dividing the group of separated children into those who live with their father and those who live with their mother at age 16, and interacting these two experiences with the father’s education, it appears that parental separation only has greater effects for children of higher-educated fathers if they live with their mother. Likewise, the father’s education only amplifies the effects of parental separation if the nonresident parent (in 86.5% of the cases the father) does not pay child maintenance. Additional analysis (available upon request) also found more heterogeneity in effects across levels of paternal education for children who had little to no contact with their father compared to children who did see their father at least from time to time/had occasional contact with their father.

Instead of being interested in the interaction effect of the education of individual parents, one could also be interested in the overall (educational) resources the parents provide. One possibility is to take the average of both parents’ school-leaving age, as in Model 6, or to use a ‘dominance’ measure indicating the highest level of education within the parental union, as in Model 7. Both models show that the likelihood to attain tertiary education decreases more for children with higher-educated parents that separated compared to those that have lower-educated parents who separated. The interaction with the ‘average’ measure is smaller and not statistically significant, probably due to a higher weight the measure gives to maternal education relative to the dominance measure.

Another way to operationalize parents’ education is to create categorical measures of possible combinations of both parents’ education, to single out the most and least advantaged groups of families. In Model 8 of Table 4, a measure that indicates who in the couple has attained at least more than high school education is used as a moderator of the effects of parental separation. The model shows that children whose parents both have more than high school education have an almost double as large separation penalty compared to offspring without more than high school educated parents.^4^

**Table 4:** Various types of models explaining the attainment of tertiary education at age 30

	Model 8: LPM (basic model)		Model 9: logistic regression (OR)		Model 10: multiple imputation		Model 11: multiple imputation	
	Coef.	SE	OR	SE	Coef.	SE	Coef.	SE
Parental separation								
Parental separation before age 16	−0.068**	0.01	0.48**	0.08	−0.061**	0.01		
Parental separation between ages 5 and 16							−0.039**	0.01
Parental resources								
Only mother more than high school	0.111**	0.02	2.05**	0.21	0.111**	0.02	0.070**	0.02
Only father more than high school	0.098**	0.01	1.91**	0.17	0.095**	0.01	0.053**	0.01
Both more than high school education	0.341**	0.01	5.67**	0.39	0.338**	0.01	0.260**	0.01
Interaction effects								
Par sep × Mother more than high school	−0.010	0.03	1.30	0.32	−0.039	0.04	−0.050	0.04
Par sep × Father more than high school	0.005	0.03	1.41	0.31	−0.007	0.03	0.003	0.03
Par sep × Both more than high school	−0.059*	0.03	1.22	0.23	−0.062*	0.03	−0.076**	0.03
Pre-separation variables								
Material resources							0.023**	0.00
Maternal psychological well-being							−0.014**	0.00
Cognitive ability at age 5							0.102**	0.01
Behavioural problems at age 5							−0.014	0.01
Constant	0.141**	0.01	0.165	0.01	0.143**	0.01	0.145**	0.01
Adjusted *R* ^2^	0.109							
*N*	10,254		10,254		11,211		11,211	

† *p* < 0.10; * *p* < 0.05; ** *p* < 0.01; data from British Cohort Study 1970

### Absolute Versus Relative Differences in Probabilities

**Fig. 1:**
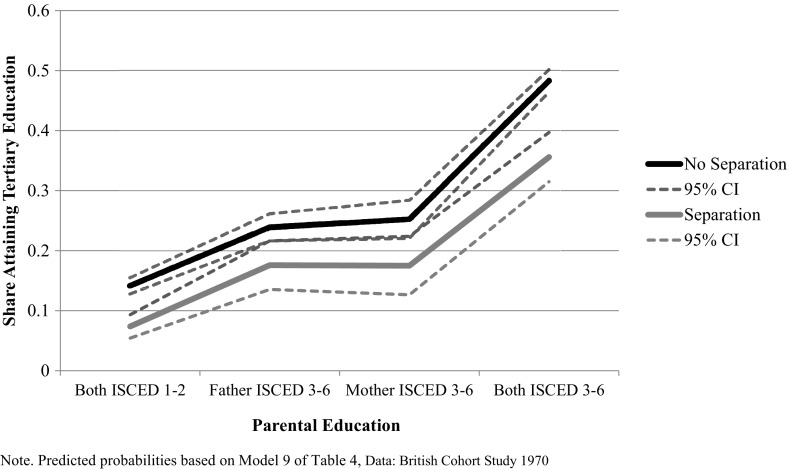
Predicted probabilities of attaining tertiary education by parental education (based on logistic regression)

So far, we have presented our results on tertiary attainment based on absolute differences in probabilities, using Linear Probability Models (LPM). In order to test whether results change if we consider relative differences, we also estimated a logistic regression, whose estimates in terms of odds ratios are reported in model 9. In the case of odds ratios, the interaction effects between parental separation and parental education are not estimated precisely (i.e. they are not statistically significant) and go in opposite directions compared to those of the previous models. If we were to focus on odds ratios, we should acknowledge that no firm conclusion can be made about heterogeneity in the effect of parental separation by parental education or even that children of higher-educated parents seem to be less affected by parental separation (if we were to focus only on the sign of the coefficient). Substantive conclusions are thus different if we consider absolute and relative differences in educational attainment. This can also be seen if one considers predicted probabilities derived from the Logistic Regression of Model 9, as plotted in Fig. 1.^5^ We can observe identical absolute differences between separated and non-separated families compared to the LPM model (Model 8), i.e. the absolute gap in tertiary educational attainment is bigger for children of higher-educated parents. At the same time, the relative gap is bigger for children of parents without educational qualifications as also found for Model 9 when using odds ratios. The probability of attaining tertiary education for children of lower-educated parents who separated is half the probability of their counterparts from non-separated families, whereas for children from separated higher-educated families the probability is three-quarters the probability for children from intact higher-educated families.

Models 10 and 11 look at the robustness of the results to multiple imputation of missing data and the inclusion of pre-separation controls. The results indicate that the effects reported in the basic model are robust to such variations in the methodological choices made. Methods dealing with attrition based on imputation might not account fully for the influence of non-random missing data (Young and Johnson 2015). We therefore looked at the first wave of a different data source to obtain estimates that are not affected by attrition (even though non-response at the moment of interview remains). Table 5 displays the results when estimated with the Understanding Society data. Results are robust to applying this different data source, providing some additional evidence that in this particular case attrition does not affect substantive conclusions. It also reduces concerns that data quality plays an essential role for the use of these two data sets.

**Table 5:** LPM models explaining the attainment of tertiary education by birth cohort using Understanding Society Data

	Model 12 Full sample including sample weights	
	Coef.	SE
Parental separation		
Parental separation before age 14	−0.066**	0.02
Parental resources		
Only mother more than high school	0.203**	0.01
Only father more than high school	0.156**	0.01
Both more than high school education	0.334**	0.01
Interaction effects		
Par div × Mother more than high school	−0.015	0.04
Par div × Father more than high school	0.057	0.04
Par div × Both more than high school	−0.062*	0.03
Constant	0.231**	0.01
*N*	22,248	

Sample weights included. † *p* < 0.10;* *p* < 0.05; ** *p* < 0.01; data from Understanding Society

### Operationalization of Other Variables

Table 6 displays variation in results based on the operationalization of childhood family structure and educational attainment. First, we operationalized family structure as living with both biological parents at age 16, which did not affect results. This suggests that when looking at single parenthood in general, similar conclusions are reached as when looking at parental separation in particular.^6^ In additional analysis, not shown, we excluded children that lived with a step-parent at age 16 from the group of children that have experienced parental separation, and the results were robust.

**Table 6:** OLS/LPM models explaining educational attainment variables by parental separation

	Model 13 Attainment of tertiary education		Model 14 Age left full-time education		Model 15 Transition to post-compulsory education age 16	
	Coef.	SE	Coef.	SE	Coef.	SE
Parental separation						
Parental separation before age 16			−0.439**	0.07	−0.076**	0.02
Not living with both parents at age 16	−0.049**	0.01				
Parental separation ‘placebo’ age 17 and 19					0.006	0.05
Parental resources						
Only mother more than high school	0.105**	0.03	0.650**	0.11	0.161**	0.02
Only father more than high school	0.098**	0.02	0.555**	0.08	0.147**	0.02
Both more than high school education	0.266**	0.02	2.308**	0.08	0.411**	0.01
Interaction effects						
Par sep × Mother more than high school	0.005	0.03	0.144	0.30	0.008	0.04
Par sep × Father more than high school	−0.001	0.03	−0.127	0.15	−0.032	0.03
Par sep × Both more than high school	−0.075**	0.02	−0.624**	0.17	−0.067*	0.03
Placebo × Mother more than high school					0.031	0.10
Placebo × Father more than high school					−0.000	0.10
Placebo × Both more than high school					−0.003	0.07
Constant	0.138**	0.01	16.84**	0.04	0.287**	0.01
Adjusted *R* ^2^	0.108		0.115		0.128	
*N*	10,254		10,254		10,254	

† *p* < 0.10; * *p* < 0.05; ** *p* < 0.01; data from British Cohort Study 1970

Second, Models 14 and 15 show how robust the results are to operationalizing educational attainment of the respondent. Both a continuous measure of educational attainment (age left full-time education) and a lower cut-off of educational attainment (transiting to post-compulsory secondary education at age 16) provide similar results.

### Endogeneity

For the final step in our analysis, we monitor the possible influence of endogeneity on our results. Table 6 displays the results of our ‘placebo test’ where parental separations that took place between ages 17 and 19 are regressed on making the transition to post-compulsory education at age 16. What can be observed is that parental separations between ages 17 and 19 are not associated to having previously made the transition to post-compulsory secondary education (*A*-levels). This suggests that relatively stable preexisting unobserved factors do not produce (heterogeneous) associations of parental separation with educational attainment (see Bernardi and Boertien 2016). At the same time, we do observe in Model 15 that parental separations that occurred before age 16 are (heterogeneously) related to educational attainment. The interpretation inferred from these two observations combined would be that the heterogeneous associations of parental separation with making the transition to post-compulsory education are not caused by relatively stable unobserved characteristics.^7^

## Discussion

Research on the effects of parental separation on child outcomes has attempted to answer the question of whether children from higher socio-economic backgrounds are affected to a larger extent by separation than others. On the one hand, socio-economically advantaged parents might have the resources to prevent their children from being negatively affected by separation, but on the other hand children from advantaged families might also lose more from a less smooth access to resources of their parents caused by parental separation. Studies on the topic to date have not been able to give a clear answer to this question, with some studies finding that those from higher socio-economic backgrounds experience a higher ‘separation penalty’ (Bernardi and Radl 2014; Biblarz and Raferty 1993; Martin 2012; McLanahan and Sandefur 1994) and other studies finding the opposite (Albertini and Dronkers 2009; Augustine 2014; Cavanagh and Huston 2006; Grätz 2015; Mandemakers and Kalmijn 2014).

This variability in results can be driven by differences across countries, the children’s outcomes considered and identification strategies used. In this study, we investigated to what extent this variation in results could be due to methodological choices made. We focused on one outcome (educational attainment), one birth cohort (1970) and one country (Britain). Firstly, we looked at the influence of operationalizing parental background. Our results pointed out that the distinction between maternal and paternal resources is essential. Whereas maternal education dampens the effects of separation, paternal education amplifies the effects of separation (when entered into one model as separate measures).

Additional analysis supported the explanation that these patterns are primarily driven by lost access to the father’s resources (because he normally moves out), and therefore, the more resources the father has, the more resources the child loses. This analysis showed that the effects of parental separation do not differ according to the father’s education for children residing with their father or children receiving child maintenance payments (who hence still have smooth access to the nonresident parent’s financial resources). This observation fits with earlier research that found that children’s success becomes more dependent on the resident parents’ characteristics, normally the mother’s, following parental separation (Beller 2009; Erola and Jalovaara 2012). From that perspective, the results of this research area also fit with the hypothesis that parental separation complicates the transmission of human capital within families (Coleman 1988). The transmission between the nonresident parent and children becomes less smooth following parental separation.

Indeed highly educated mothers seem able to reduce the negative implications of separation for their children (although in this case the estimates were highly imprecise). At the same time, the overall effect of paternal resources seems to dominate over the compensating effect of maternal resources, as the combined measures of both parents’ education in general point at the more resources a family has, the larger the effects of separation. This conclusion might differ depending on the gender of the child, an issue we did not look at in this paper. It could be that access to nonresident parents’ resources differs according to the gender of the child, an issue for future research.

Second, we pinpointed a methodological choice that appeared to be highly influential when using a dichotomous dependent variable, namely whether to report effect sizes in relative or absolute terms. In this paper, we showed that when estimated as absolute differences in terms of probabilities, children of higher-educated parents experience a larger ‘separation penalty’, whereas in terms of relative differences no heterogeneity in the effects of parental separation was observed, and the sign of coefficients even went in the opposite direction. Other methodological choices such as the operationalization of educational attainment of children, addressing issues of endogeneity, looking at the effects of single parenthood in general instead of just parental separation, and different ways of treating missing data appeared to be less influential in our case.

In summary, the analysis of this study suggests that methodological choices made when estimating heterogeneity in the effects of parental separation can be consequential to the conclusions drawn. When one looks at heterogeneity by maternal resources and measures the inequality in children’s outcomes in terms of odds ratios, no differences in the effects of parental separation between advantaged and disadvantaged families are observed. On the other hand, if one looks at paternal resources and estimates marginal effects or predicted probabilities, it appears that advantaged children are affected more negatively by parental separation than disadvantaged children. The knowledge gained from these findings should reduce future research possibilities of ‘shopping’ for a measure or comparison that will lead to a statistically significant or desired result (Gelman and Loken 2014). Future studies could present results both in absolute and relative terms, and should monitor the influence of both mother’s and father’s resources, as not taking account one of these resources will only tell part of the story.

If we return to the review of the literature presented in the beginning of the article, we see that quite some variation across studies could actually reflect differences in these choices made rather than actual differences across periods, outcomes and countries. The distinction between maternal and paternal resources could realign many of the inconsistent findings of earlier studies. Studying Table 1, one notices that 11 out of the 13 papers reviewed are, broadly speaking, consistent with a story of paternal resources amplifying separation effects and maternal resources dampening them. At the same time, two of these 11 studies find that when paternal resources are not taken into account, greater effects of parental separation on children’s outcomes are observed for children of higher-educated mothers (Albertini and Dronkers 2009; Augustine 2014). In our paper, while not controlling for education of the father, no heterogeneity in the effects of parental separation was found. That Albertini and Dronkers (2009) did find heterogeneity in effects could be due to their choice of methods based on odds ratios, which as mentioned, are more likely to lead to greater effects for disadvantaged children. The other paper (Augustine 2014) focused on cognitive ability, which could therefore be a possible source for their slightly differing results compared to the other studies considered.

The remaining two papers that do not fit the general pattern outlined above are those by Grätz (2015) and by Cavanagh and Huston (2006). These studies look at different countries (e.g. Germany for Grätz, most other studies are on the USA or the UK) or a combination of cohort, country and outcome not investigated by other studies (Cavanagh and Huston 2006). It is therefore likely that, also when making the same methodological choices, heterogeneity in the effects of parental separation differs across contexts and outcomes studied. This point was also proven in the comparative study of Bernardi and Radl (2014). Future research could concentrate on these substantive differences in effects, but should thus ensure that methodological and operational choices made are harmonized across studies.

The final question to be answered is, do children from higher social backgrounds indeed have more to lose from a parental separation, or do high-resource parents also manage to compensate for the experience of disadvantage in the case of separation? For the 1970 British birth cohort, the answer seems to be mostly the former. Clear patterns of heterogeneity emerged when looking at the effects of parental separation on educational attainment, where children whose fathers are higher-educated displayed larger ‘separation penalties’. The same is found when looking at combined measures of parental education. That this result is absent when paternal education is not taken into account, but only maternal education is, therefore seems more an issue of having an incomplete measure of parental resources, and should thus be avoided. Overall, these results lend support to the argument that parental separation can complicate the transmission of advantage across generations (Coleman 1988) and makes this transmission more dependent on the mother’s characteristics (Beller 2009; Erola and Jalovaara 2012). But the complication in access to the father’s resources might only be relevant for educational attainment, an outcome that appears relatively responsive to parental income (Bernardi and Boertien 2016). In addition to this qualification, it remains to be seen whether the results obtained in this paper also hold in other countries or periods. In other contexts, educational attainment could be less dependent on parental resources, or access to fathers’ resources could be easier due to higher levels of father involvement or joint custody.

## Footnotes

1 There is relatively little knowledge about what explains socio-economic differentials in divorce risk. A recent study in the UK found that home ownership, parental divorce, step-children and unemployment explain most of the differences in divorce risk according to women’s educational levels (Boertien and Härkönen 2014). These would then be important variables to include as controls.

2 At age 16, students can make the transition to A-levels, an academic track usually required to enter tertiary education.

3 We checked for multicollinearity in this model but found no cause for concern as the highest Variance Inflation Factor found was 2.26 for the main effect of the mother´s education.

4 We also employed a different classification of parental education distinguishing between those who have two parents with tertiary education, and those whose parents, neither father nor mother, have no tertiary education. The biggest separation penalty is observed in children whose father is the only parent to have tertiary education (not shown). But only a small percentage of all children belong to the group with both parents having tertiary education. We also looked at how results differ when using parental occupation (manual vs non-manual jobs of parents), and results were similar to the categorical education measure presented in Table 3.

5 Linear Probability Models and Logistic Regression using Odds Ratios differ on more points than just reporting results in an absolute or relative way. As the results of Fig. 1 show, also when calculating predicted probabilities based on logistic regressions, we observe the same results as observed for the LPM model. This excludes the possibility that it is the underlying estimation method that causes differences in results between Models 8 and 9 of Table 4, but rather the presentation of results in absolute or relative terms.

6 Which does not, however, imply that the effects of different family transitions such as parental death and parental separation are the same, rather that our results are not sensitive to the choice of operationalization of the family structure variable.

7 The assumption here is that selection into parental separation according to parental education does not differ by age of the children, which appeared to hold when looking at differences on a set of observables by age at separation in analysis presented elsewhere (Bernardi and Boertien 2016).
